# Relationship between rectal bleeding and radiation delivery time in intensity-modulated radiotherapy for localized prostate cancer: a preliminary study

**DOI:** 10.1093/jrr/rrag055

**Published:** 2026-07-21

**Authors:** Nozomi Kita, Natsuo Tomita, Taiki Takaoka, Dai Okazaki, Masanari Niwa, Akira Torii, Seiya Takano, Masanosuke Oguri, Hikaru Tsuzuki, Machiko Ukai, Masahiro Sasaguchi, Takahiro Tsuchiya, Takahiro Yasui, Akio Hiwatashi

**Affiliations:** Department of Radiology, Nagoya City University Graduate School of Medical Sciences, 1 Kawasumi, Mizuho-cho, Mizuho-ku, Nagoya, Aichi 467-8601, Japan; Department of Radiology, Nagoya City University Graduate School of Medical Sciences, 1 Kawasumi, Mizuho-cho, Mizuho-ku, Nagoya, Aichi 467-8601, Japan; Department of Radiology, Nagoya City University Graduate School of Medical Sciences, 1 Kawasumi, Mizuho-cho, Mizuho-ku, Nagoya, Aichi 467-8601, Japan; Department of Radiology, Nagoya City University Graduate School of Medical Sciences, 1 Kawasumi, Mizuho-cho, Mizuho-ku, Nagoya, Aichi 467-8601, Japan; Department of Radiology, Nagoya City University Graduate School of Medical Sciences, 1 Kawasumi, Mizuho-cho, Mizuho-ku, Nagoya, Aichi 467-8601, Japan; Department of Radiology, Nagoya City University Graduate School of Medical Sciences, 1 Kawasumi, Mizuho-cho, Mizuho-ku, Nagoya, Aichi 467-8601, Japan; Department of Radiology, Nagoya City University Graduate School of Medical Sciences, 1 Kawasumi, Mizuho-cho, Mizuho-ku, Nagoya, Aichi 467-8601, Japan; Department of Radiology, Nagoya City University Graduate School of Medical Sciences, 1 Kawasumi, Mizuho-cho, Mizuho-ku, Nagoya, Aichi 467-8601, Japan; Department of Radiology, Nagoya City University Graduate School of Medical Sciences, 1 Kawasumi, Mizuho-cho, Mizuho-ku, Nagoya, Aichi 467-8601, Japan; Department of Radiology, Nagoya City University Graduate School of Medical Sciences, 1 Kawasumi, Mizuho-cho, Mizuho-ku, Nagoya, Aichi 467-8601, Japan; Department of Radiology, Nagoya City University Graduate School of Medical Sciences, 1 Kawasumi, Mizuho-cho, Mizuho-ku, Nagoya, Aichi 467-8601, Japan; Department of Radiology, Nagoya City University Graduate School of Medical Sciences, 1 Kawasumi, Mizuho-cho, Mizuho-ku, Nagoya, Aichi 467-8601, Japan; Department of Urology, Nagoya City University Graduate School of Medical Sciences, 1 Kawasumi, Mizuho-cho, Mizuho-ku, Nagoya, Aichi 467-8601, Japan; Department of Radiology, Nagoya City University Graduate School of Medical Sciences, 1 Kawasumi, Mizuho-cho, Mizuho-ku, Nagoya, Aichi 467-8601, Japan

**Keywords:** prostate cancer, intensity-modulated radiotherapy

## Abstract

The present study investigated the relationship between late grade ≥ 2 rectal bleeding and radiation delivery time in intensity-modulated radiotherapy (IMRT) for localized prostate cancer. We analyzed patients with localized prostate cancer treated with IMRT at our institution between July 2012 and February 2018, including only those treated with 74.8 Gy in 34 fractions (i.e. a fraction dose of 2.2 Gy). To evaluate the relationship between risk factors and late grade ≥ 2 rectal bleeding, we performed univariate analyses using the Gray test and the Fine-Gray model, and multivariate analysis using the Fine-Gray model. Additionally, propensity score matching was performed to adjust for confounding factors. A total of 142 cases were analyzed. The median follow-up period was 83 months. The 7-year cumulative incidence of late grade ≥ 2 rectal bleeding for the entire cohort was 5.7%. The group with shorter delivery time (≤150 s) had a significantly lower 7-year cumulative incidence of late grade ≥ 2 rectal bleeding compared to the group with longer delivery time (>150 s) (1.5% vs. 9.7%, *P* = 0.036). Multivariate analysis showed a trend toward an association between longer delivery time and increased late grade ≥ 2 rectal bleeding (hazard ratio 6.1, 95% confidence interval 0.78–48, *P* = 0.085). In the propensity score-matched cohort, the shorter delivery time group showed a significantly lower incidence of late grade ≥ 2 rectal bleeding (0% vs. 11.1%, *P* = 0.024). In IMRT for localized prostate cancer, shorter radiation delivery time may reduce the incidence of late grade ≥ 2 rectal bleeding.

## INTRODUCTION

Radiation therapy for localized prostate cancer is an established treatment modality. The widespread adoption of intensity-modulated radiotherapy (IMRT) and image-guided radiotherapy (IGRT) has improved treatment accuracy. However, rectal bleeding remains a significant side effect of radiation therapy for prostate cancer. The introduction of spacers, such as SpaceOAR (Boston Scientific, Marlborough, MA, USA), inserted between the rectum and prostate, has significantly reduced the incidence of grade ≥ 1 rectal bleeding [1]. Despite these advances, it is challenging to insert spacers in all cases, and some patients decline spacer use, underscoring the importance of analyzing factors contributing to rectal bleeding.

We previously analyzed cases of localized prostate cancer treated with IMRT at a fractional dose of 2.2 Gy, and observed a significant difference in the incidence of late grade ≥ 2 rectal bleeding between the Tomotherapy and volumetric modulated arc therapy (VMAT) groups [2]. While differences in the energy used were considered a potential cause, the exact reason remained unclear. In the present study, we hypothesized that the radiation delivery time may contribute to rectal bleeding because prostate shifts during radiation therapy for prostate cancer and similarly rectal movement during radiation therapy for rectal cancer have both been well documented [3–6]. Recent studies using magnetic resonance imaging-guided radiotherapy (MRgRT) have confirmed even more precise movements [7, 8]. Even if the target position is corrected using IGRT immediately before irradiation, as the duration of radiation delivery increases, the range of intrafraction movement of the prostate and rectum may also increase. This discrepancy may result in higher radiation doses being delivered to the rectum than planned, potentially leading to rectal bleeding. As a preliminary analysis, the present study investigated the relationship between late grade ≥ 2 rectal bleeding and radiation delivery time in IMRT for localized prostate cancer.

## MATERIALS AND METHODS

### Study population

We herein examined patients with localized prostate cancer who were treated at our institution with Tomotherapy or VMAT using a dose of 74.8 Gy in 34 fractions. Inclusion criteria were as follows: (i) T1-T3N0M0 clinical stage; (ii) adenocarcinoma confirmed through a histological examination; (iii) treatment with Tomotherapy or VMAT; (iv) a fraction dose of 2.2 Gy. Exclusion criteria were as follows: (i) dose-volume histogram (DVH) data unavailable; (ii) a rectal evaluation not feasible due to prior rectal surgery; (iii) incomplete IMRT; (iv) dose fractionation of 72.6 Gy in 33 fractions (this dose fractionation was used for primarily low-risk cases in our institution). The present study was approved by our Institutional Review Board (No. 60-24-0100). Since this study was conducted retrospectively, the requirement for written informed consent was waived, and an opt-out form was provided on the website for those who did not wish to participate. The present study followed the ethical standards laid down in the 1964 Declaration of Helsinki and its later amendments.

### Treatment

Details on the treatment plan have been described in previous studies [2, 9]. IMRT was delivered using 6 MV X-rays with Tomotherapy and 10 MV X-rays with VMAT from a linear accelerator. The clinical target volume (CTV) was contoured to include the entire prostate and seminal vesicles according to the T stage. In the T1 stage, one-third of seminal vesicles were included in the CTV, 50% in the T2 stage and 100% in the T3 stage. Planning target volume (PTV) margins for CTV were set at 7–8 mm anteriorly, 5–6 mm posteriorly, 7–8 mm cranio-caudally and 6–7 mm laterally. The superior border of the rectum was defined as 1 cm above the PTV, and the inferior border as 1 cm below the PTV. Dose constraints for the target and organs at risk were common across both modalities and have been previously described [10, 11]. In all groups, a dose of 74.8 Gy in 34 fractions was prescribed to the Dmean of the PTV. Tomotherapy typically utilized a 2.5-cm dynamic jaw and a pitch of 0.43, while VMAT was delivered using 2 arcs. Daily pre-treatment alignment was performed using megavoltage CT (MVCT) for Tomotherapy and cone-beam CT (CBCT) for VMAT. The superposition algorithm was used for dose calculation in both Tomotherapy and VMAT plans. All cases underwent IMRT without the use of spacers.

As a principle, patients in the high- and intermediate-risk groups received 6 months of neoadjuvant androgen deprivation therapy (ADT) prior to radiation therapy. Additionally, high-risk patients received 2 years of adjuvant ADT.

### Follow-up, endpoint and dose–volume parameters

Follow-up evaluations were conducted every 1 to 3 months until 1 year after IMRT, and subsequently every 3 to 6 months. The follow-up period was calculated from the start date of IMRT. Late adverse events were defined as those occurring three months or later after the initiation of IMRT. Adverse events were assessed according to the Common Terminology Criteria for Adverse Events, version 5.0. The primary endpoint was late grade ≥ 2 rectal bleeding.

DVH data and dose distributions were analyzed using RayStation (RaySearch Medical Laboratories AB, Stockholm, Sweden). Rectum Vx_Gy_ (%) was defined as the percentage of the rectal volume receiving at least x Gy of the prescribed radiation dose. To evaluate rectal doses across different intensity levels, the following parameters were extracted from the DVH: V71.1_Gy_ (%) (corresponding to V95_%_) as the high-dose region parameter, and V57.7_Gy_ (%) and V38.5_Gy_ (%) as the intermediate- and low-dose region parameters, respectively. The latter two values were selected based on the specific dose constraints applied at our institution at the time of treatment.

### Statistical analysis

To examine the relationship between risk factors and late grade ≥ 2 rectal bleeding, univariate analyses were performed using the Gray test and the Fine-Gray model, with death as a competing risk. Multivariate analysis was conducted using the Fine-Gray model, with death as a competing risk. Additionally, propensity score matching was performed to adjust for potential confounding factors. Propensity scores were calculated using a logistic regression model incorporating age, anticoagulant therapy and rectal dose parameters and one-to-one matching was performed between the two groups (radiation delivery time ≤ 150 s vs. >150 s). Statistical analyses were conducted using EZR, a graphical user interface for R (The R Foundation for Statistical Computing, Vienna, Austria) [12].

## RESULTS

A total of 169 patients met the inclusion criteria. Treatments were administered between July 2012 and February 2018. One patient each was excluded due to the unavailability of DVH data, the lack of a rectal evaluation and IMRT not being completed. In addition, 24 patients were excluded because they were treated with 72.6 Gy in 33 fractions, resulting in 142 patients treated with 74.8 Gy in 34 fractions being analyzed in this study. The median follow-up period was 83 months (range, 10–132). Of 142 patients, 66 were in the Tomotherapy group and 76 in the VMAT group. The overall median radiation delivery time was 151 s (range, 74–411), and it was significantly longer in the Tomotherapy group than in the VMAT group (median 217 s [range, 128–411] vs. 150 s [range, 74–154]; *P* < 0.001). Seven-year overall survival (OS), biochemical relapse-free survival (bRFS), and clinical relapse-free survival (cRFS) rates were 93.1% (95% confidence interval [CI], 86.5–96.6), 87.6% (95% CI, 79.9–92.4) and 89.7% (95% CI, 82.3–94.1), respectively. Seven-year late grade ≥ 2 rectal bleeding and late grade ≥ 2 genitourinary (GU) toxicity rates were 5.7% (95% CI, 2.7–10.4) and 10.3% (95% CI, 5.7–16.5), respectively.

Table 1 shows patient and treatment characteristics. Median age was 73 years (range, 52–83). Twenty-five patients (18%) were receiving anticoagulant therapy. When patients were stratified into two groups based on the median delivery time (≤150 s vs. >150 s), significant differences were observed in Rectum V71.1_Gy_ and Rectum V57.7_Gy_ (*P* < 0.001 and *P* = 0.004, respectively). No significant differences were found in other characteristics. Regarding the treatment modalities, the ≤150 s group consisted of 4 cases (6%) of Tomotherapy and 66 cases (94%) of VMAT, whereas the >150 s group consisted of 62 cases (86%) of Tomotherapy and 10 cases (14%) of VMAT.

**Table 1:** Patient and treatment characteristics

Characteristics	All patients	Delivery time	Delivery time	*P*-value
		≤150 s group	>150 s group	
	*n* = 142	*n* = 70	*n* = 72	
Age (years)	73 (52–83)	73 (52–83)	72 (53–82)	0.96
≤73	78 (55%)	40 (57%)	38 (53%)	0.62
>73	64 (45%)	30 (43%)	34 (47%)	
PSA (ng/mL)	10.7 (3.5–373.3)	10.1 (4.0–373.3)	11.5 (3.5–88.9)	0.16
T-stage				0.87
T1c/T2a	29 (20%)/43 (30%)	13 (19%)/24 (34%)	16 (22%)/19 (26%)	
T2b/T2c	11 (7.7%)/26 (18%)	5 (7.1%)/14 (20%)	6 (8.3%)/12 (17%)	
T3a/T3b	21 (15%)/12 (8.5%)	9 (13%)/5 (7.1%)	12 (17%)/7 (9.7%)	
Gleason score				0.13
6	16 (11%)	8 (11%)	8 (11%)	
7	79 (56%)	44 (63%)	35 (49%)	
8–10	47 (33%)	18 (26%)	29 (40%)	
Risk				0.23
Low	1 (0.7%)	0 (0%)	1 (1.4%)	
Intermediate	55 (39%)	31 (44%)	24 (33%)	
High	86 (61%)	39 (56%)	47 (65%)	
Anticoagulants	25 (18%)	13 (19%)	12 (17%)	0.83
Radiation dose (Gy)	74.8 (74.8–74.8)	74.8 (74.8–74.8)	74.8 (74.8–74.8)	1
Rectum V71.1_Gy_ (%)	4.5 (0.02–7.9)	3.4 (0.02–7.6)	5.2 (0.8–7.9)	<0.001
Rectum V57.7_Gy_ (%)	15.4 (7.6–21.0)	14.7 (9.6–21.0)	16.0 (7.6–18.7)	0.004
Rectum V38.5_Gy_ (%)	30.4 (18.6–42.5)	30.0 (21.8–38.3)	30.9 (18.6–42.5)	0.072
Delivery time (s)	151 (74–411)	150 (74–150)	215 (151–411)	<0.001

Rectum Vx_Gy_ (%), the percentage of the rectal volume receiving at least x Gy of the prescribed radiation dose.

Figure 1 shows the incidence of late grade ≥ 2 rectal bleeding in the group with a delivery time of ≤150 s and the group with >150 s. The 7-year cumulative incidence of late grade ≥ 2 rectal bleeding was 1.5% in the ≤150 s group and 9.7% in the >150 s group (*P* = 0.036). Regarding GU toxicity, late grade ≥ 2 hematuria occurred in only 4 cases, and no significant difference was observed between the ≤150 s and > 150 s groups (*P* = 0.22). Similarly, no significant association was observed between these two groups and overall late grade ≥ 2 GU toxicity (*P* = 0.51). Table 2 presents the results of univariate and multivariate analyses for late grade ≥ 2 rectal bleeding. Although a significant difference was observed in the intergroup comparison stratified by radiation delivery time using the Gray test (*P* = 0.036), the univariate analysis using the Fine-Gray model showed borderline significance (*P* = 0.069). In the multivariate analysis, although no factors reached statistical significance, anticoagulant therapy (hazard ratio [HR] 4.3, 95% CI 0.90–20, *P* = 0.069) and radiation delivery time (HR 6.1, 95% CI 0.78–48, *P* = 0.085) showed trends toward an association.

**Fig. 1:**
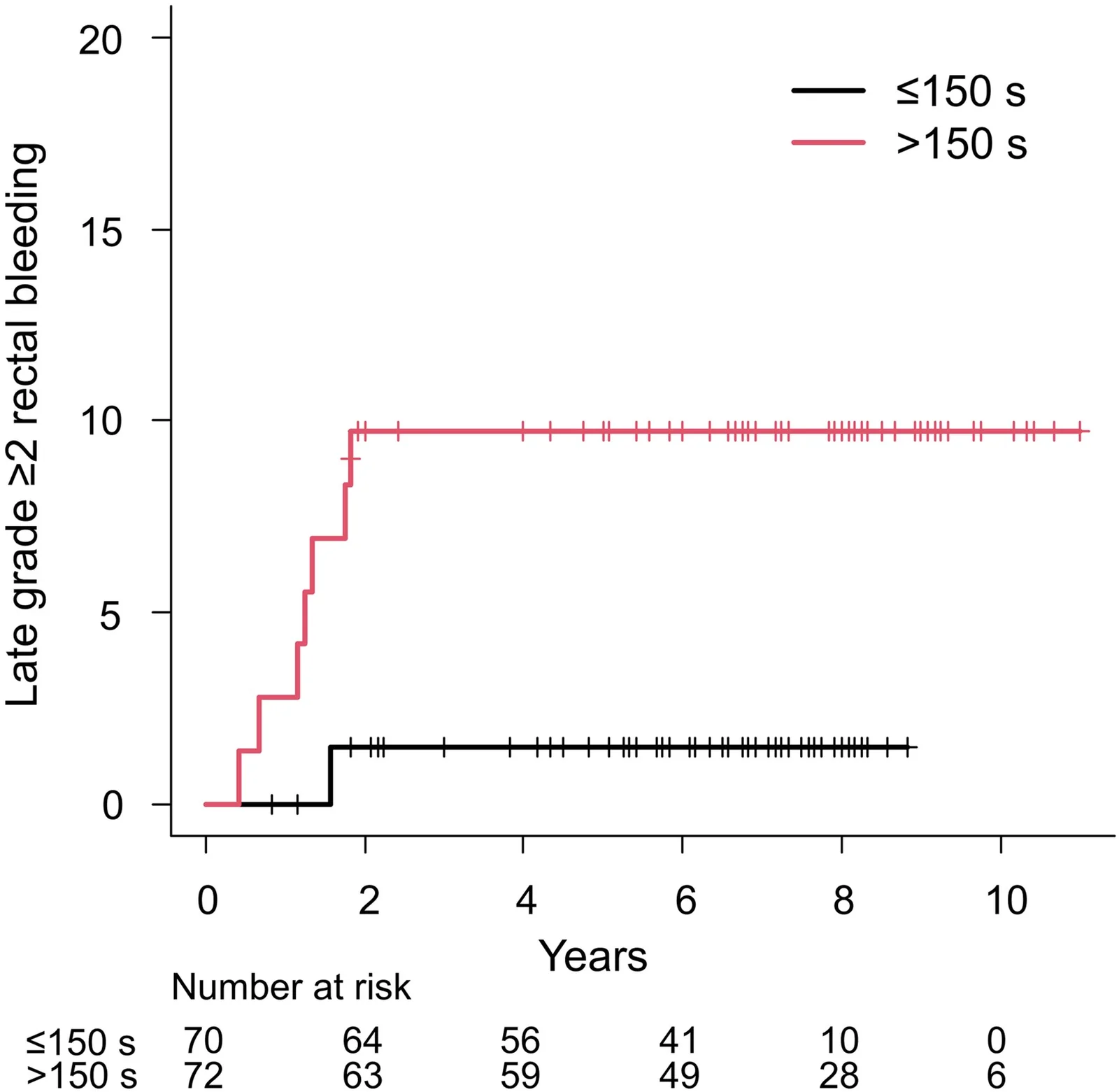
Differences in the cumulative incidence of grade ≥ 2 rectal bleeding according to radiation delivery time.

**Table 2:** Univariate and multivariate analysis of risk factors for late grade ≥ 2 rectal bleeding

Factors	Univariate analysis			Multivariate analysis		
	HR	95% CI	*P*-value	HR	95% CI	*P*-value
Age (years) (>73 vs. ≤73)	0.73	0.18–3.0	0.67	0.53	0.12–2.4	0.42
Anticoagulants (Yes vs. No)	3.1	0.74–13	0.12	4.3	0.90–20	0.069
Delivery time (s) (>150 vs. ≤150)	6.9	0.86–55	0.069	6.1	0.78–48	0.085
Rectum V71.1_Gy_ (%) (continuous)	1.3	0.94–1.9	0.10	1.1	0.62–1.9	0.79
Rectum V57.7_Gy_ (%) (continuous)	1.2	0.99–1.5	0.065	1.2	0.77–1.8	0.46
Rectum V38.5_Gy_ (%) (continuous)	1.1	0.91–1.3	0.37	1.0	0.83–1.2	0.99

HR, hazard ratio; 95% CI, 95% confidence interval; Rectum Vx_Gy_ (%), the percentage of the rectal volume receiving at least x Gy of the prescribed radiation dose.

Propensity score matching was performed to adjust for potential confounding factors associated with rectal bleeding, including age, anticoagulant therapy and rectal dose parameters of V71.1_Gy_, V57.7_Gy_ and V38.5_Gy_. Table 3 shows the characteristics of the matched cohorts (≤150 s vs. >150 s). A total of 90 patients were selected, with 45 patients in each group. There were no significant differences in these matched characteristics between the two groups. Regarding the treatment modalities in the matched cohort, the ≤150 s group consisted of 4 cases (9%) of Tomotherapy and 41 cases (91%) of VMAT, whereas the >150 s group consisted of 36 cases (80%) of Tomotherapy and 9 cases (20%) of VMAT. In the propensity score-matched cohort, the 7-year cumulative incidence of late grade ≥ 2 rectal bleeding was 0% in the ≤150 s group and 11.1% in the >150 s group, indicating a significant difference (*P* = 0.024) (Fig. 2).

**Table 3:** Patient and treatment characteristics after propensity score matching for factors related to late grade ≥ 2 rectal bleeding

Characteristics	Delivery time	Delivery time	*P*-value
	≤150 s group	>150 s group	
	*n* = 45	*n* = 45	
Age (years)	73 (52–83)	74 (54–82)	0.65
≤73	26 (58%)	22 (49%)	0.53
>73	19 (42%)	23 (51%)	
PSA (ng/mL)	10.3 (4.0–373.3)	11.2 (3.5–74.1)	0.66
T-stage			0.40
T1c/T2a	9 (20%)/14 (31%)	10 (22%)/12 (27%)	
T2b/T2c	5 (11%) /9 (20%)	4 (8.9%)/6 (13%)	
T3a/T3b	3 (6.7%)/5 (11%)	10 (22%)/3 (6.7%)	
Gleason score			0.058
6	6 (13%)	3 (6.7%)	
7	29 (64%)	23 (51%)	
8–10	10 (22%)	19 (42%)	
Risk			0.28
Low	0 (0%)	0 (0%)	
Intermediate	21 (47%)	15 (33%)	
High	24 (53%)	30 (67%)	
Anticoagulants	6 (13%)	10 (22%)	0.41
Radiation dose (Gy)	74.8 (74.8–74.8)	74.8 (74.8–74.8)	1
Rectum V71.1_Gy_ (%)	4.4 (0.02–7.6)	4.6 (0.8–6.5)	0.88
Rectum V57.7_Gy_ (%)	15.5 (10.3–21.0)	15.0 (7.6–18.4)	0.65
Rectum V38.5_Gy_ (%)	30.3 (21.8–38.3)	30.7 (18.6–38.4)	0.90
Delivery time (s)	150 (75–150)	215 (151–411)	<0.001

Rectum Vx_Gy_ (%), the percentage of the rectal volume receiving at least x Gy of the prescribed radiation dose.

**Fig. 2:**
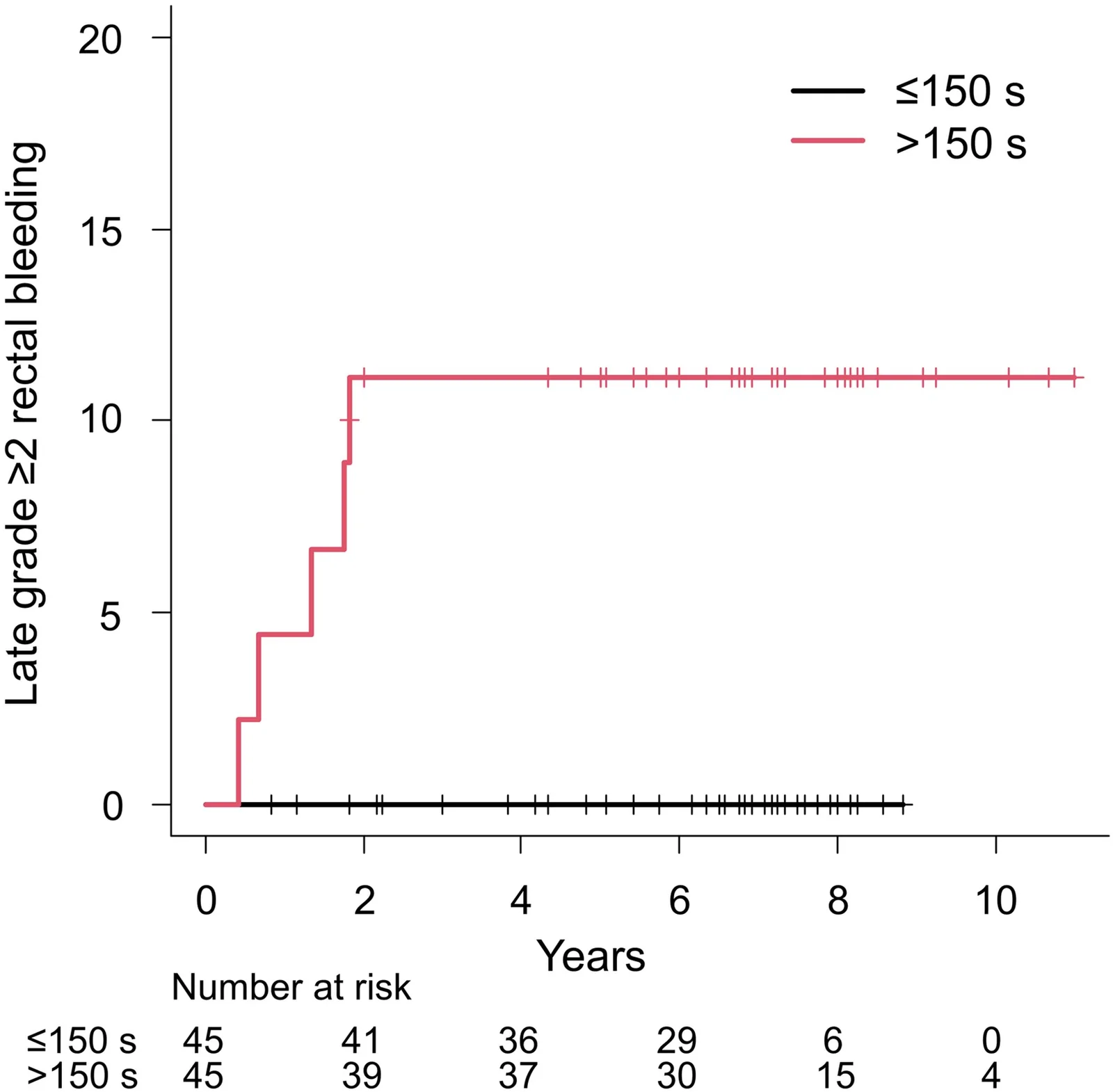
Differences in the cumulative incidence of grade ≥ 2 rectal bleeding according to radiation delivery time in the propensity score-matched cohort.

## DISCUSSION

We investigated the relationship between late grade ≥ 2 rectal bleeding and radiation delivery time in IMRT for localized prostate cancer. The 7-year late grade ≥ 2 rectal bleeding rate was 5.7%. The group with shorter delivery time ≤ 150 s had a significantly lower cumulative incidence of late grade ≥ 2 rectal bleeding than the group with longer delivery time > 150 s (1.5% vs. 9.7%, respectively, *P* = 0.036). The multivariate analysis showed that longer radiation delivery time tended to increase the risk of late grade ≥ 2 rectal bleeding (HR 6.1, 95% CI 0.78–48, *P* = 0.085). Although the difference did not reach statistical significance, likely due to the limited sample size, a trend was observed suggesting that shorter radiation delivery time might be associated with a reduced incidence of late grade ≥ 2 rectal bleeding. Consequently, our preliminary analysis weakly suggests a potential link between delivery time and rectal toxicity, indicating that this factor warrants further investigation in a larger cohort.

Real-time monitoring of organ movement during radiation therapy was previously challenging; however, with the introduction of MRgRT, it has become possible. Previous studies reported that the prostate moves ~3–4 mm during irradiation with MRgRT [7,13–15]. Additionally, the prostate was shown to move by ~2 mm on CT scans taken before each treatment session [16]. While numerous studies have focused on prostate movement during radiation therapy for localized prostate cancer, very few have examined rectal movement in this setting. In radiation therapy for rectal cancer, the rectum was shown to move by ~3–6 mm during irradiation [17–19]. Displacements of ~2–6 mm of the rectum were confirmed in cone beam CT images acquired at each treatment session [20, 21].

Although research on rectal cancer demonstrated that the rectum moves by ~3–6 mm during irradiation, many studies suggest that the degree of rectal movement is not substantial. However, in radiation therapy for localized prostate cancer, the dose distribution often shows a steep dose gradient on the rectal side. Without the use of spacers, even a slight anterior shift of the rectum may result in higher radiation doses being delivered to it. In the present study, we aligned the positions of the prostate and rectum using CT before irradiation; however, with longer treatment durations, we anticipate that peristaltic motion of the rectum may increase uncertainty in the dose delivered to the rectum. Although the wide confidence intervals in our multivariate analysis warrant cautious interpretation due to the limited sample size, the observed trend offers a valuable clinical signal that shorter delivery time may reduce rectal toxicity. Nevertheless, the clinical implications of even a modest reduction in rectal toxicity are substantial. Therefore, despite the preliminary nature of these findings, we believe there is value in further verifying this hypothesis through larger, prospective studies. While VMAT provides stable and shorter delivery times, Tomotherapy is associated with longer and more variable durations. Moreover, this disparity is accentuated when considering the entire process from the start of image guidance to the completion of irradiation. At our institution, MVCT for Tomotherapy requires ~90–120 s, whereas CBCT for VMAT takes only about 30 s. However, Radixact, the latest version of the Tomotherapy system, has incorporated ClearRT, which has significantly reduced IGRT acquisition time to ~30 s [22]. Considering these cumulative time differences, our results suggest that in Tomotherapy planning, optimizing for shorter delivery times without compromising dose-volume constraints may be a crucial strategy to reduce late rectal toxicity.

The multivariate analysis suggested a trend toward an increased risk of late grade ≥ 2 rectal bleeding associated with the use of anticoagulants (*P* = 0.069), although it did not reach statistical significance. In this study, the 7-year cumulative risk among patients using anticoagulants was 12.2%. Previous studies also reported an increased risk of late rectal bleeding associated with anticoagulant use [23–25]. Therefore, we propose closer follow-ups of localized prostate cancer patients receiving radiation therapy while on anticoagulants, particularly in cases where a spacer is not inserted.

Seven-year OS, bRFS and cRFS rates were 93.1%, 87.6% and 89.7%, respectively. Since the majority of analyzed subjects were categorized as intermediate- or high-risk, these outcomes may be regarded as favorable. Previous studies on external beam radiation therapy (EBRT) for localized prostate cancer indicated that 5-year bRFS rates for intermediate- and high-risk patients ranged from ~80 to 95% [26–28]. Regarding adverse effects, the results of the present study may also be regarded as favorable. Seven-year late grade ≥ 2 rectal bleeding and late grade ≥ 2 GU rates were 5.7% and 10.3%, respectively. Although these rates vary across reports depending on factors such as the use of SpaceOAR and the treatment era, previous studies on EBRT for localized prostate cancer have reported that the cumulative incidence of late grade ≥ 2 gastrointestinal toxicity ranges from 1% to 12%, while that of late grade ≥ 2 GU toxicity ranges from 4% to 25% [26–31]. The rates observed in the present study were consistent with these values.

This study has several limitations. As a single-institution retrospective study, there is a potential for bias. A multicenter study with a larger sample size is needed to validate the trends observed in this initial analysis. Additionally, radiation delivery time may reflect differences in treatment modalities; therefore, the possibility that modality-specific dosimetric characteristics influenced the results cannot be completely ruled out. Furthermore, current treatment for localized prostate cancer often involves a fraction dose of 3 Gy. The results of this study, which analyzed cases treated with a fraction dose of 2.2 Gy, may differ. At our institution, we currently treat localized prostate cancer with a fraction dose of 3 Gy, and we plan to analyze these data in the future. In addition, radiation therapy for localized prostate cancer is now commonly performed after placing a spacer between the prostate and rectum. The use of a spacer significantly reduces the amount of radiation delivered to the rectum, thereby lowering the incidence of rectal bleeding. However, this study is expected to be useful for patients who refuse spacer placement.

In conclusion, we investigated the relationship between late grade ≥ 2 rectal bleeding and radiation delivery time in IMRT for localized prostate cancer. Among 142 patients treated with IMRT at a dose of 74.8 Gy in 34 fractions, the 7-year cumulative incidence of late grade ≥ 2 rectal bleeding was 5.7%. The multivariate analysis suggested that shorter radiation delivery time may reduce the incidence of late grade ≥ 2 rectal bleeding. In the propensity score-matched cohort, the group with shorter radiation delivery time showed a significantly lower incidence of late grade ≥ 2 rectal bleeding. These preliminary findings warrant further verification through larger, prospective studies.
